# Association between *BRAF* (V600E) mutation and clinicopathological features of papillary thyroid carcinoma: a Brazilian single-centre case series

**DOI:** 10.20945/2359-3997000000120

**Published:** 2019-03-18

**Authors:** Danielle Pessôa-Pereira, Mateus Fernandes da Silva Medeiros, Virna Mendonça Sampaio Lima, Joaquim Custódio da Silva, Taíse Lima de Oliveira Cerqueira, Igor Campos da Silva, Luciano Espinheira Fonseca, Luiz José Lobão Sampaio, Cláudio Rogério Alves de Lima, Helton Estrela Ramos

**Affiliations:** 1 Universidade Federal da Bahia Universidade Federal da Bahia Instituto de Saúde e Ciência Laboratório de Estudo da Tireoide Salvador BA Brasil Departamento de Biorregulação, Laboratório de Estudo da Tireoide, Instituto de Saúde e Ciência, Universidade Federal da Bahia (UFBA), Salvador, BA, Brasil; 2 Hospital São Rafael Departamento de Anatomia Patológica e Citopatologia Salvador BA Brasil Departamento de Anatomia Patológica e Citopatologia, Hospital São Rafael, Salvador, BA, Brasil; 3 Universidade Federal da Bahia Universidade Federal da Bahia Faculdade de Medicina da Bahia Departamento de Anatomia Patológica e Medicina Legal Salvador BA Brasil Departamento de Anatomia Patológica e Medicina Legal, Faculdade de Medicina da Bahia, Universidade Federal da Bahia (UFBA), Salvador, BA, Brasil; 4 Hospital São Rafael Departamento de Medicina Nuclear Salvador BA Brasil Departamento de Medicina Nuclear, Hospital São Rafael, Salvador, BA, Brasil; 5 Hospital São Rafael Departamento de Cirurgia de Cabeça e Pescoço Salvador BA Brasil Departamento de Cirurgia de Cabeça e Pescoço, Hospital São Rafael, Salvador, BA, Brasil

**Keywords:** Papillary thyroid cancer, *BRAF* mutation, Hashimoto's thyroiditis

## Abstract

**Objectives:**

We aimed to investigate the prevalence of the *BRAF* (V600E) mutation in consecutive cases of papillary thyroid carcinoma (PTC) in patients diagnosed and treated at the Hospital Sao Rafael (Salvador, BA, Brazil) and evaluate its association with clinical and pathological characteristics of PTC.

**Subjects and methods:**

We retrospectively enrolled in the study a total of 43 consecutive PTC patients who underwent total thyroidectomy. We performed DNA extraction from formalin-fixed paraffin-embedded (FFPE) tumour tissue samples. Polymerase chain reaction (PCR) and direct sequencing were used to determine *BRAF* (V600E) mutation status. Univariate and multivariate logistic regression analyses were employed to identify independent associations.

**Results:**

The prevalence of *BRAF* (V600E) mutation was 65.1% (28/43). A high frequency of older patients (p value: 0.004) was observed among the *BRAF*-mutated PTC group and, in contrast, a low frequency of concurrent Hashimoto's thyroiditis (HT) (p value: 0.011) was noted. Multivariate analysis confirmed that older age (OR: 1.15; 95% CI: 1.00 – 1.33; p value: 0.047) and HT (OR: 0.05; 95% CI: 0.006-0.40; p value: 0.005) were independent factors associated with *BRAF* (V600E) mutation.

**Conclusion:**

We found a high prevalence of *BRAF* (V600E) mutation in PTC cases. Older age and no concurrent HT were independently associated with *BRAF* (V600E) mutation.

## INTRODUCTION

Thyroid cancer is the most frequently diagnosed endocrine malignancy worldwide, currently ranking in ninth place for global incidence (1). Papillary thyroid carcinoma (PTC), which accounts for up to 85% of all thyroid cancer cases (2), has a relatively indolent behaviour and shows better prognosis than other malignant thyroid tumours, such as medullary and anaplastic thyroid carcinoma (3). However, some patients may experience extrathyroidal extension (ETE), local and/or distant metastases, as well as present recurrent disease after surgery and radioactive iodine therapy, all of which significantly contribute to a poorer prognosis (4). Recurrence and mortality risk stratifications, which are mainly based on clinical and pathological criteria, are currently the main tool for determining suitable clinical management of PTC (5,6). Nevertheless, those clinicopathological factors, such as older age, histological subtypes, tumour size and distant metastasis, have not been able to provide complete accuracy in terms of predicting a poor prognosis (7). Therefore, many studies have focused on identifying additional parameters, such as molecular markers, in order to provide an accurate risk assessment.

Significant advances in understanding oncogenic events involved in the onset and progression of PTC have arisen over recent decades. Thyroid oncogenesis often involves constitutive activation of the mitogen-activated protein kinase (MAPK) kinase (MEKK)/extracellular signal-regulated kinase (ERK) pathway, usually driven by the T1799A somatic mutation in the v-raf murine sarcoma viral oncogene homolog B1 (*BRAF*) exon 15, a process which results in a V600E amino acid replacement (8). Several studies have shown a positive association between *BRAF* (V600E) and PTC aggressive phenotype, including ETE, distant metastasis, and silencing of thyroid-specific iodine-metabolizing genes (9). On the other hand, these findings have not been found in some other studies (10), raising doubts about whether *BRAF* (V600E) could be used as an appropriate prognostic factor for PTC.

Considering the lack of consistent evidence in the literature and that there are no published reports on the prevalence of *BRAF* (V600E) mutation in PTC patients in North-eastern Brazil, we conducted a cross-sectional study of consecutive case series of PTC from a reference hospital in Salvador, BA, Brazil, in order to evaluate the association between *BRAF* (V600E) mutation and clinicopathological features of PTC.

## SUBJECTS AND METHODS

### Ethics statement

This study was formally approved by the institutional Research Ethics Committee of the Federal University of Bahia (n. 102.290), along with the medical board of Sao Rafael Hospital (Salvador, Bahia, Brazil), and was carried out in accordance with the Declaration of Helsinki of the World Medical Association.

### Patient selection

We retrospectively enrolled 43 consecutive PTC patients who had undergone total thyroidectomy as initial treatment for PTC in the Department of Head and Neck Surgery at Sao Rafael Hospital between 2006 and 2012. All clinical and pathological data, including age, sex, tumour size, histological subtype, multifocality, concurrent Hashimoto's thyroiditis (HT), ETE, vascular invasion, lymph node and distant metastasis status, were collected from medical records (Table 1). Patients were classified as low, intermediate and high risk according to the 2015 American Thyroid Association (ATA) risk stratification for well-differentiated thyroid cancer (6,11), as well as according to the Brazilian consensus (5). We excluded from the study patients whose cancer-related medical records and/or formalin-fixed paraffin-embedded (FFPE) tumour specimens derived from their surgical resections were unavailable.

**Table 1:** Clinical and pathological features of the PTC patients

Patient	Age (y)	Sex	Tumour size (mm)	PTC variant	HT	Multifocal disease	ETE	Vascular invasion	LM	Distant metastasis	8th AJCC TNM				2015 ATA Recurrence Risk	*BRAF* (V600E) mutation
											T	N	M	CS		
1	20	F	25	Classical	Yes	Yes	No	Yes	No	No	2	0	0	I	Intermediate	Negative
2	12	F	40	Classical	Yes	Yes	No	No	Yes	No	2	1b	0	I	High	Negative
3	17	F	6	Classical	No	No	No	No	Yes	No	1a	1b	0	I	Intermediate	Negative
4	17	F	10	Classical	No	No	No	No	No	No	1a	0	0	I	Low	Positive
5	13	F	22	Classical	Yes	Yes	No	No	No	No	2	0	0	I	Low	Negative
6	36	F	25	Follicular	Yes	Yes	No	No	No	No	2	0	0	I	Low	Negative
7	40	F	20	Follicular	No	Yes	No	No	No	Yes	1b	0	1	II	High	Positive
8	26	F	15	Classical	Yes	Yes	Yes	No	Yes	No	1a	1b	0	I	Intermediate	Positive
9	44	F	10	Classical	Yes	No	No	No	Yes	No	1a	1a	0	I	Low	Negative
10	38	F	12	Classical	Yes	Yes	No	No	No	No	1b	0	0	I	Low	Positive
11	39	F	12	Classical	Yes	Yes	No	No	No	No	1b	0	0	I	Low	Negative
12	26	F	10	Classical	No	No	No	No	Yes	No	1a	1a	0	I	Low	Positive
13	40	M	13	Classical	No	Yes	No	No	No	No	1b	0	0	I	Low	Positive
14	31	F	30	Follicular	No	No	No	No	No	No	2	0	0	I	Low	Negative
15	45	F	15	Classical	No	No	Yes	No	Yes	No	1b	1a	0	I	Intermediate	Positive
16	36	F	10	Follicular	No	No	No	No	Yes	No	1a	1a	0	I	Low	Negative
17	41	F	5	Classical	Yes	Yes	No	No	Yes	No	1a	1a	1	I	Low	Negative
18	45	F	20	Classical	No	Yes	No	No	No	No	1b	0	0	I	Low	Positive
19	32	F	16	Classical	Yes	No	No	No	No	No	1b	0	0	I	Low	Negative
20	36	F	13	Follicular	No	No	No	No	No	Yes	1b	0	1	II	High	Negative
21	35	F	15	Tall cell	Yes	No	No	No	Yes	No	1b	1a	0	I	High	Negative
22	30	F	15	Follicular	No	Yes	No	No	Yes	No	1b	1a	0	I	Intermediate	Negative
23	24	F	10	Classical	No	No	No	No	No	No	1a	0	0	I	High	Positive
24	37	F	20	Classical	No	Yes	No	No	No	No	1b	0	0	I	High	Positive
25	42	M	15	Classical	No	No	No	No	No	No	1b	0	0	I	Low	Positive
26	33	F	20	Classical	Yes	No	Yes	No	Yes	No	3b	1a	0	I	High	Positive
27	29	F	10	Classical	No	No	No	No	No	No	1a	0	0	I	Low	Positive
28	27	F	20	Classical	No	Yes	No	No	No	No	1b	0	0	I	Low	Positive
29	24	F	20	Classical	Yes	No	No	No	No	No	1b	0	0	I	Low	Negative
30	41	M	35	Classical	No	No	No	No	No	No	2	0	0	I	Low	Positive
31	46	F	10	Follicular	No	Yes	No	No	Yes	No	1a	1a	0	I	Low	Positive
32	46	F	37	Classical	No	Yes	No	Yes	Yes	No	2	1a	0	I	Intermediate	Positive
33	46	F	9	Classical	No	Yes	No	No	No	No	1a	0	0	I	Low	Positive
34	61	F	50	Trabecular	No	Yes	No	No	No	No	3a	0	0	II	Low	Positive
35	42	F	30	Classical	Yes	No	No	No	Yes	No	2	1b	0	I	Low	Positive
36	62	F	50	Classical	Yes	Yes	No	Yes	No	No	3a	0	0	II	Intermediate	Positive
37	44	F	15	Oncocytic	No	Yes	No	No	No	No	1b	0	0	I	Low	Positive
38	28	F	15	Classical	No	No	No	No	No	No	1b	0	0	I	Low	Positive
39	41	F	13	Classical	No	Yes	No	No	Yes	No	1b	1a	0	I	Low	Positive
40	36	F	13	Classical	No	No	No	No	No	No	1b	0	0	I	Low	Positive
41	78	F	15	Follicular	No	Yes	No	No	No	No	1b	0	0	I	Low	Positive
42	69	F	18	Classical	Yes	Yes	Yes	No	No	No	3b	0	0	II	High	Positive
43	38	F	18	Classical	Yes	No	No	No	Yes	No	1b	1a	0	I	High	Positive

Y: years-old; F: female; M: male; mm: millimetres; PTC: papillary thyroid carcinoma; HT: Hashimoto's thyroiditis; ETE: extrathyroidal extension; AJCC: American Joint Committee on Cancer; CS: clinical stage; ATA: American Thyroid Association.

### Tumor samples

FFPE blocks were obtained from the archives of the Department of Anatomic Pathology and Cytopathology at Sao Rafael Hospital in order to acquire thyroid tumour samples for *BRAF* (V600E) mutation analysis. All samples were histologically reviewed on haematoxylin and eosin (HE)-stained slides by two pathologists. Tumours were staged according to the 8^th^ edition of the TNM-based staging system proposed by the American Joint Association on Cancer (AJCC). Representative tumour areas containing at least 60% of cancer cells were marked on the HE-stained sections. Subsequently, five sequential sections of 10-μm thickness were obtained from each chosen FFPE tissue block. Marked tumour areas were manually scraped off from the unstained sections by sterile needles using their respective HE-stained slides as a guide. In cases of multifocal disease, we only collected the largest tumour focus for analysis.

### DNA isolation

Genomic DNA isolation from the FFPE samples was performed using Gentra Puregene Core Kit B (Qiagen, Valencia, CA, USA) at the Thyroid Study Laboratory (Salvador, BA, Brasil), preceded by a total removal of the paraffin from the samples with xylene and washing steps with ethanol, according to the manufacturer's protocol. Concentration levels and quality were assessed using a Nano Spectrophotometer Kasvi K23-0002 (Kasvi, Sao Jose dos Pinhais, PR, Brazil).

### Polymerase chain reaction

Polymerase chain reaction (PCR) was performed to amplify the exon 15 of *BRAF* from the isolated DNA, as previously described (8). Briefly, *BRAF* exon 15 was amplified in a 20-μl reaction volume containing 100 ng of genomic DNA, 7.5 pmol of each primer (Forward: 5’-AAACTCTTCATAATGCTTGCTCTG-3’; Reverse: 5’-GGCCAAAAATTAATCAGTGGA-3’), 100 μm deoxynucleoside triphosphates (dNTPs), 5 μCi [α^32^P]dCTP, 1.5 mm MgCl_2_, Platinum TaqDNA polymerase high fidelity and buffer (Thermo Fisher Scientific, Waltham, MA, USA). The PCR reaction was heated to 95 °C for 5 minutes for initial denaturation followed by 35 cycles of 95 °C for 30 seconds, 58 °C for 30 seconds and 72 °C for 45 seconds, with a final extension at 72 °C for 5 minutes. All PCR amplifications were performed using the Veriti 96-Well Thermal Cycler (Thermo Fisher Scientific, Waltham, MA, USA).

### Agarose gel eletrophoresis

PCR products were electrophoresed in 1,5% UltraPure^TM^ Agarose diluted in UltraPure^TM^ TBE Buffer 1X (Thermo Fisher Scientific, Waltham, MA, USA) containing 0,1-μl/mL SYBR Safe DNA Gel Stain (Thermo Fisher Scientific, Waltham, MA, USA) at 90v for 45 minutes using the Loccus horizontal gel electrophoresis system, model LCH 13×15 (Loccus Biotechnology, Cotia, SP, Brazil). *BRAF* exon 15 amplification was confirmed by the presence of single bands containing 231 base pairs visualized using a Safe Imager 2.0 (Thermo Fisher Scientific, Waltham, MA, USA).

### DNA sequecing

**Figure 1:**
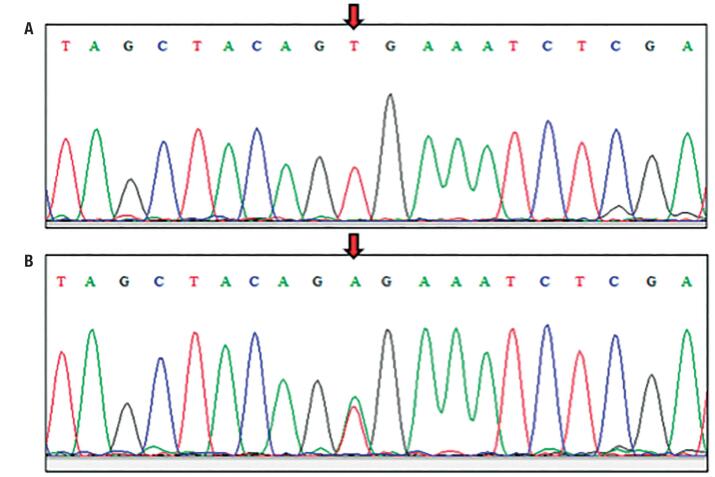
Representative DNA sequencing of *BRAF* exon 15. A) Absence of thymine to adenine replacement (T1799A), indicating a wild-type *BRAF*. B) Heterozygous *BRAF* (V600E) mutation.

After confirmation of *BRAF* exon 15 amplification, PCR products were purified using the PureLink Quick PCR Purification Kit (Thermo Fisher Scientific, Waltham, MA, USA) and precipitated using 70% isopropyl alcohol and Hi-DITM Formamide (Thermo Fisher Scientific, Waltham, MA, USA). Subsequently, DNA sequencing reaction were performed using a BigDye^®^ Terminator v3.1 Cycle Sequencing Kit (Thermo Fisher Scientific, Waltham, MA, USA) in the capillary automatic sequencer ABI 3130XL PRISM Genetic Analyzer (Thermo Fisher Scientific, Waltham, MA, USA) at the Fundação Oswaldo Cruz – Instituto Gonçalo Moniz (Salvador, Bahia, Brazil). The *BRAF* (V600E) mutation was confirmed by comparing sequences using the Basic Local Alignment Search Tool (BLAST) program, available at the NCBI website (http://www.ncbi.nlm.nih.gov/BLAST/). We considered tumours as being *BRAF* (V600E) positive those which were harbouring a homozygous or heterozygous *BRAF* (V600E) mutation (T1799A) (Figure 1).

### Statistical analysis

Qualitative variables were presented using frequencies and percentages, whereas continuous variables were presented using mean values and standard deviation. An independent *t*-test was used to compare the mean values of continuous variables and a chi-square test or Fisher's exact test to compare frequencies of qualitative variables. Univariate and multivariate logistic regression analyses were performed to evaluate the associations between clinical and pathological parameters of PTC and the *BRAF* (V600E) mutation. Values of odds ratio (OR) were determined to measure the strength of the associations. The statistical significance of these associations was based on p value < 0.05 and 95% confidence intervals (CI). All statistical data analyses were performed using the statistical software program SPSS for Windows, v. 20.0 (IBM, Armonk, NY, USA).

## RESULTS

### Clinical and pathological characteristics of PTC patients

All clinical and pathological data of the 43 selected PTC patients are summarized in Table 2. The mean age at diagnosis was 37 years, ranging from 12 to 78 years. Even though only four patients (3.6%) were considered as paediatric cases (≤ 18 years old), most patients were > 55 years old (90.7%). Therefore, our case series were mainly comprised by early-stage PTC patients (88.4% and 11.6% for clinical stage I and II, respectively), according to current pathological TNM system. Most patients were female (40/43; 93%) and presented multifocal disease (23/43; 53.5%). Seventeen patients (39.5%) had concurrent Hashimoto's thyroiditis (HT). As expected, there were more conventional PTC cases (32/43; 8/43; 74.4%), followed by the follicular variant (8/43; 18.6%). Three cases of rare PTC histological subtypes were observed, which included an oncocytic (oxyphilic), variant, a trabecular variant and a tall cell variant. Tumour sizes ranged from 5 to 50 mm (mean, 19.23 mm), most of them having more than 10 mm (32/43; 74.4%). Furthermore, 16 patients (37.2%) had lymph node metastasis at diagnosis, but only few cases presented vascular invasion (3/43; 7%), ETE (4/43; 9.3%) and distant metastases (2/43; 2%). As stated by 2015 ATA risk stratification, 27 (62.8%) and 16 patients had low and intermediate/high risk of recurrence, respectively. Moreover, 24 (55.8%) and 19 (44.2%) patients had very low/low and intermediate/high risk of recurrence, respectively, according to the current Brazilian consensus.

**Table 2:** Associations between *BRAF* (V600E) mutation and clinicopathological features of PTC

Characteristics		All patients	*BRAF* mutation (-)	*BRAF* mutation (+)	p value
Total number of patients (%)		43	15	28	−
Paediatric patients (≤ 18 y)		4 (9.3)	3	1 (3.6)	
Age at diagnosis, y					
	Mean	37.04 ± 13.58	29.73 ± 10.18	40.96 ± 13.69	**0.004**
	Median	37			
	Range	12-78			
	< 55	39 (90.7)	15 (100)	24 (85.7)	0.280^a^
	≥ 55	4 (9.3)	0	4 (14.3)	
Sex					0.540^a^
	Male	3 (7)	0	3 (10.7)	
	Female	40 (93)	15 (100)	25 (89.3)	
Tumour size, mm					
	Mean	19.23 ± 11.36	17.60 ± 9.51	19.21 ± 11.20	0.622
	Median	15			
	Range	5-50			
	< 10	11 (25.6)	2 (13.3)	1 (3.6)	0.275^a^
	≥ 10	32 (74.4)	13 (86.7)	27 (96.4)	
Histological subtype					0.150
	Classical PTC	32 (74.4)	9 (60)	23 (82.1)	
	Non-classical PTC	11 (25.6)	6 (40)	5 (17.9)	
	Follicular variant	8 (18.6)	5 (33.3)	3 (10.7)	
	Tall cell variant	1 (3.6)	1 (6.7)	0	
	Trabecular variant	1 (3.6)	0	1 (3.6)	
	Oncocytic variant	1 (3.6)	0	1 (3.6)	
Hashimoto's thyroiditis					**0.011**
	No	26 (60.5)	5 (33.3)	21 (75)	
	Yes	17 (39.5)	10 (66.7)	7 (25)	
Multifocal disease					0.54
	No	20 (46.5)	8 (53.3)	12 (42.9)	
	Yes	23 (53.5)	7 (46.7)	16 (57.1)	
ETE					0.280^a^
	No	39 (90.7)	15 (100)	24 (85.7)	
	Yes	4 (9.3)	0	4 (14.3)	
Vascular invasion					1^a^
	No	40 (93)	14 (93.3)	26 (92.9)	
	Yes	3 (7)	1 (6.7)	2 (7.1)	
Lymph node metastasis					0.348
	No	27 (62.8)	8 (53.3)	19 (67.9)	
	Yes	16 (37.2)	7 (46.7)	9 (32.1)	
Distant metastasis					1^a^
	No	41 (95.3)	14 (93.3)	27 (96.4)	
	Yes	2 (4.7)	1 (6.7)	1 (3.6)	
8th AJCC/TNM stage system					0.643^a^
	I	38 (88.4)	14 (93.3)	24 (85.7)	
	II	5 (11.6)	1 (6.7)	4 (14.3)	
2015 ATA recurrence risk					0.782
	Low	27 (62.8)	9 (60)	18 (64.3)	
	Intermediate/High	16 (37.2)	6 (40)	10 (35.7)	
SBEM recurrence risk					1
	Very low/Low	24 (55.8)	9 (60)	16 (57.1)	
	Intermediate/High	19 (44.2)	6 (40)	12 (42.9)	

a Fisher's exact test. Y: years-old; mm, millimetres; ETE: extrathyroidal extension; AJCC: American Joint Committee on Cancer; ATA: American Thyroid Association; SBEM: *Sociedade Brasileira de Endocrinologia e Metabologia.*

### Association of *BRAF* (V600E) mutation and clinicopathological characteristics of PTC

*BRAF* (V600E) mutation was detected in 28 PTC tumour samples (65.1%). No homozygous mutant or other genetic alterations were found in *BRAF* exon 15. Patients were categorized according to *BRAF* (V600E) mutation status in order to evaluate possible associations between clinical and pathological features of PTC and presence of *BRAF* (V600E) (Table 2). All ≥ 55-year-old (4/43) and male (3/43) patients presented PTC harbouring a *BRAF* (V600E) mutation. Only one paediatric patient (1/3) had a *BRAF*-mutant PTC: a 17-year-old young woman who presented PTC with no aggressive behaviour. Furthermore, follicular variant was predominantly observed in the *BRAF*-wild type PTC group (5/8; 62.5%). *BRAF* (V600E) was detected in the isolated rare cases of trabecular variant and oncocytic variant, but not in the tall cell PTC variant. All patients who presented PTC with ETE (4/43) were tested as *BRAF* (V600E) positives.

After comparing the groups, we found that the frequency of *BRAF* (V600E) mutation was significantly higher in older patients (40.96 ± 13.69 vs 29.73 ± 10.18; p value: 0.004) and lower in PTC with concurrent HT (25 vs 66.7%; p value: 0.011). However, no significant differences were found for other clinical and pathological parameters, including intermediate to high risk of recurrence.

### Univariate and multivariate logistic regression analysis

To further investigate the effect of *BRAF* (V600E) on age and HT in PTC patients, we performed a univariate analysis to measure the association between these parameters and the presence of a *BRAF* (V600E) mutation (Table 3). Once again, we found that older age at diagnosis (OR: 1.09; 95% CI: 1.02-1.17; p value: 0.016) and negative HT (OR: 0.17; 95% CI: 0,04-0,66; p value: 0.011) were significantly associated with *BRAF*-mutated PTC. Finally, we performed a multivariate regression logistic analysis in order to investigate whether these features could be considered as independent predictors of *BRAF* (V600E) mutation. Crude OR values confirmed the significant associations revealed in the univariate analysis. After adjusting for tumour size, multifocality, vascular invasion, extrathyroidal extension, lymph node and distant metastasis, we found that older age (OR: 1.15; 95% CI: 1.00-1.33; p value: 0.047) and negative HT (OR: 0.05; 95% CI: 0.006-0.40; p value: 0.005) were still significantly associated with the *BRAF* (V600E) mutation.

**Table 3:** Univariate and multivariate regression logistic analysis of BRAF (V600E) mutation and clinicopathological features of PTC

Characteristics		Univariate			Multivariate		
		OR (95% CI)	p value	OR (95% CI)	p value	Adjusted OR^a^ (95% CI)	p value
Age at diagnosis, y		1		1		1	
	Mean	1.09 (1.02 – 1.17)	**0.016**	1.09 (1.01 – 1.18)	**0.021**	1.15 (1.00 – 1.33)	**0.047**
Hashimoto's thyroiditis							
	No	1		1		1	
	Yes	0.17 (0.04 – 0.66)	**0.011**	0.14 (0.03 – 0.68)	**0.014**	0.05 (0.006 – 0.40)	**0.005**

Y: years; OR: odds ratio.

a Adjusted for tumour size, multifocality, vascular invasion, extrathyroidal extension, lymph node and distant metastasis.

## DISCUSSION

Although PTC usually displays excellent behaviour at clinical presentation (3), some cases may present a worse prognosis, with ETE, lymph node and distant metastases, as well as acquiring treatment resistance (4). In an effort to find potential prognostic biomarkers in PTC that could identify those patients, several studies have investigated the role of the *BRAF* (V600E) mutation, a common genetic alteration in PTC. However, it remains unclear whether *BRAF* (V600E) is closely associated with aggressive behaviour of PTC. Because few studies have been performed by using Brazilian cohorts, this research was designed to verify the frequency of *BRAF* (V600E) mutation in PTC patients in a reference hospital in Salvador, BA, Brazil, and investigate its association with their clinical and pathological characteristics.

The prevalence rates of the *BRAF* (V600E) mutation in PTC range between 27.3 to 90.2% worldwide (12,13). Most Brazilian studies that investigated the presence of *BRAF* mutation in FFPE or fresh frozen PTC samples were conducted in the South-eastern region, mainly in the State of Sao Paulo (14–18). While these studies reported *BRAF* (V600E) frequencies between 28.1 to 48.3%, we noted a higher prevalence rate (65.1%) in PTC patients in the present study. Our finding is similar to those reported in PTC patients diagnosed in cities from other Brazilian regions, such as Goiânia (74/116; 63,8%) and Porto Alegre (19/32; 59,4%) (19,20). Some studies have implied that the increased occurrence of *BRAF* (V600E) mutation in PTC may be associated with high dietary iodine intake (21). We found in a previous study from our laboratory a high risk of excessive nutritional iodine intake among schoolchildren in Salvador (22), a coastal Brazilian city. If extrapolated to the adult population, this finding suggested that most residents in Salvador may also have a relatively high iodine intake. However, considering the low frequency reported by the studies conducted in Sao Paulo, where thyroid cancer incidence and iodine intake levels are considered higher than other Brazilian regions (23), we assume there are other features that could have influenced the high prevalence rate of *BRAF* (V600E) mutation found in the present study, such as environmental factors and genetic background of the PTC patients.

In the past few decades, numerous meta-analyses have reported *BRAF* (V600E) as being strictly associated with aggressive clinicopathological features and poorer clinical outcomes PTC (12,13), but this association remains controversial. We found that *BRAF* (V600E) mutation was significantly linked with older age, with such patients being more likely to have a *BRAF*-mutated PTC according to univariate and multivariate analyses. Advanced age has been considered an important prognostic factor for PTC patients and is significantly associated with recurrence and survival (24). Interestingly, Shen and cols. have recently demonstrated in a large cohort that age is an independent risk factor for mortality in PTC patients with *BRAF* (V600E) mutation, but not in wild type *BRAF* (25). In contrast to the adult population, differentiated thyroid cancer is an uncommon event in childhood and adolescence (26). From our case series, only four PTC patients were classified as paediatric cases. As opposed to adult PTC, paediatric PTC usually displays aggressive behaviour at clinical presentation, manifested by high incidence of multifocal disease, lymph node and distant metastasis (26). In spite of that, paediatric PTC often has a better prognosis compared to adult PTC (26). Among all paediatric PTC patients enrolled in the present study, only one presented a *BRAF*-mutant PTC. Consistently, the literature has reported low frequency of *BRAF* mutations in paediatric PTC (27). Conversely, rearranged during transfection (*RET*)/PTC translocations seems to be the most frequent genetic change in those cases, which are most often associated with radiation exposure (27). Remarkably, *BRAF* (V600E) mutation, but not *RET*/PTC rearrangements, has been associated with genomic instability and consequently with decreased expression of the sodium-iodine symporter (NIS) (14,28), somewhat corroborating the better recurrence-free survival observed in paediatric PTC patients when compared to adults.

HT is an autoimmune disease that promotes inflammation and subsequently a progressive depletion of thyroid cells, which are gradually replaced by fibrosis and mononuclear infiltrate (29). Although the association between inflammation and cancer has been well established in distinct human cancers (30), the pathological and molecular connections between HT and PTC are still discussed. It has been postulated that genetic alterations promoting carcinogenesis can activate proinflammatory programs, which result in the formation of an inflammatory tumour microenvironment (31). In turn, the inflammatory cells contribute to tumour progression by inducing the expression of growth factors, proangiogenic factors, extracellular matrix-degrading enzymes, as well as by releasing reactive oxygen species into the tumour microenvironment (30). Indeed, some studies conducted using *in vitro* models have demonstrated that *RET/*PTC rearrangements and *BRAF* (V600E) mutation, the most frequent genetic events in PTC, can increase the expression of proinflammatory chemokines and cytokines, which facilitate tumour proliferation, migration and survival (32–34). However, it is worth mentioning that most studies have reported that *RET*/PTC translocations are more frequent in PTC patients with HT than in patients with PTC alone, which has a higher frequency of *BRAF* (V600E) mutation in contrast (35,36). Consistently, we found a significant and independent negative association between *BRAF* (V600E) and HT in PTC patients. Because *RET*/PTC rearrangements is also detected in HT-affected thyroid epithelial cells (35), we believe that the molecular circuits linking HT and PTC mostly do not involve *BRAF* (V600E) mutation, but preferably *RET*/PTC rearrangements.

Overall, we did not demonstrate a significant association between *BRAF* (V600E) mutation and the remaining clinicopathological characteristics of PTC, which included sex, tumour size, histological subtype, multifocality, ETE, vascular invasion, lymph node and distant metastases, as well as intermediate/high risk to recurrence according to current guidelines. There were some important limitations in our study, notably the small sample size and the predominance of early-stage patients; therefore, we did not confirm the association between *BRAF* (V600E) mutation and aggressive behaviour and poor prognosis in PTC. The 8^th^ TNM edition recently removed microscopic ETE and regional lymph node metastasis from the classification of T3 disease. In addition, the 8^th^ TNM edition changed the age at diagnosis cut-off from 45 to 55 years, downstaging a significant number of PTC patients (37). Because we selected consecutive cases, only patients classified into the stage I/II were included in the present study. However, our results are in accordance with the majority of Brazilian studies, which did not find correlation between *BRAF* mutations and PTC aggressive phenotype (15–20). In contrast, Oler and Cerutti reported that the *BRAF* (V600E) mutation was associated with tumour size, ETE, lymph node metastasis, high risk of recurrence and mortality in PTC patients (14). Many factors could explain this discrepancy, including case selection, different statistical approaches and the effect of possible confounding factors. Taking into account the conflicting results obtained from several studies addressing *BRAF* (V600E) prognostic value in PTC around the world, some authors suggest reconsidering the clinical relevance of the *BRAF* (V600E) mutation (10). Indeed, there is a possibility that this mutation may be in fact indirectly involved in tumour progression. By causing genomic instability, the *BRAF* (V600E) mutation may coexist with secondary genetic and/or epigenetic alterations (38,39), which in turn might cooperate in terms of tumour aggressiveness and even be more reliable prognostic indicators for PTC.

In conclusion, we found a high prevalence of *BRAF* (V600E) mutation in a PTC case series from patients diagnosed and treated in Salvador, BA, Brazil. Furthermore, older age and concurrent HT was independently associated with the presence and absence of the *BRAF* (V600E) mutation, respectively. We consider that further investigation is required to clarify potential mechanisms underlying the association between HT and the *BRAF* (V600E) mutation.

## References

[1] Bray F, Ferlay J, Soerjomataram I, Siegel RL, Torre L, Jemal A. Global cancer statistics 2018: GLOBOCAN estimates of incidence and mortality worldwide for 36 cancers in 185 countries. CA Cancer J Clin. 2018. 10.3322/caac.21492 30207593

[2] Lim H, Devesa SS, Sosa JA, Check D, Kitahara CM. Trends in thyroid cancer incidence and mortality in the United States, 1974--2013. JAMA. 2017;317(13):1338-48. 10.1001/jama.2017.2719 PMC8216772 28362912

[3] Noone AM, Howlader N, Krapcho M, Miller D, Brest A, Yu M, et al. (eds.). SEER Cancer Statistics Review, 1975-2015. Bethesda, MD. National Cancer Institute. Available from: https://seer.cancer.gov/csr/1975_2015/.

[4] Hirsch D, Levy S, Tsvetov G, Gorshtein A, Slutzky-Shraga I, Akirov A, et al. Long-term outcomes and prognostic factors in patients with differentiated thyroid cancer and distant metastases. Endocr Pract. 2017;23(10):1193-200. 10.4158/EP171924.OR 28704099

[5] Rosário PW, Ward LS, Carvalho GA, Graf H, Maciel RM, Maciel LM, et al.; Sociedade Brasileira de Endocrinologia e Metabologia. Thyroid nodules and differentiated thyroid cancer: update on the Brazilian consensus. Arq Bras Endocrinol Metabol. 2013;57(4):240-64. 10.1590/s0004-27302013000400002 23828432

[6] Haugen BR, Alexander EK, Bible KC, Doherty GM, Mandel SJ, Nikiforov YE, et al. 2015 American Thyroid Association Management Guidelines for Adult Patients with Thyroid Nodules and Differentiated Thyroid Cancer: The American Thyroid Association Guidelines Task Force on Thyroid Nodules and Differentiated Thyroid Cancer. Thyroid. 2016;26(1):1-133. 10.1089/thy.2015.0020 PMC4739132 26462967

[7] Papaleontiou M, Haymart MR. New insights in risk stratification of differentiated thyroid cancer. Curr Opin Oncol. 2014;26(1):1-7. 10.1097/CCO.0000000000000022 PMC4102253 24285100

[8] Kimura ET, Nikiforova MN, Zhu Z, Knauf JA, Nikiforov YE, Fagin JA. High prevalence of BRAF mutations in thyroid cancer: genetic evidence for constitutive activation of the RET/PTC-RAS-BRAF signaling pathway in papillary thyroid carcinoma. Cancer Res. 2003;63(7):1454-7. 12670889

[9] Xing M. BRAF mutation in papillary thyroid cancer: pathogenic role, molecular bases, and clinical implications. Endocr Rev. 2007;28(7):742-62. 10.1210/er.2007-0007 17940185

[10] Gandolfi G, Sancisi V, Piana S, Ciarrocchi A. Time to re-consider the meaning of BRAF V600E mutation in papillary thyroid carcinoma. Int J Cancer. 2015;137(5):1001-11. 10.1002/ijc.28976 24828987

[11] Francis GL, Waguespack SG, Bauer AJ, Angelos P, Benvenga S, Cerutti JM, et al.; American Thyroid Association Guidelines Task Force. Management Guidelines for Children with Thyroid Nodules and Differentiated Thyroid Cancer. Thyroid. 2015;25(7):716-59. 10.1089/thy.2014.0460 PMC4854274 25900731

[12] Li C, Lee KC, Schneider EB, Zeiger MA. BRAF V600E mutation and its association with clinicopathological features of papillary thyroid cancer: a meta-analysis. J Clin Endocrinol Metab. 2012;97(12):4559-70. 10.1210/jc.2012-2104 PMC3513529 23055546

[13] Liu C, Chen T, Liu Z. Associations between BRAF(V600E) and prognostic factors and poor outcomes in papillary thyroid carcinoma: a meta-analysis. World J Surg Oncol. 2016;14(1):241. 10.1186/s12957-016-0979-1 PMC5012084 27600854

[14] Oler G, Cerutti JM. High prevalence of BRAF mutation in a Brazilian cohort of patients with sporadic papillary thyroid carcinomas: correlation with more aggressive phenotype and decreased expression of iodide-metabolizing genes. Cancer. 2009;115(5):972-80. 10.1002/cncr.24118 19152441

[15] Araujo PP, Marcello MA, Tincani AJ, Guilhen AC, Morari EC, Ward LS. mRNA BRAF expression helps to identify papillary thyroid carcinomas in thyroid nodules independently of the presence of BRAFV600E mutation. Pathol Res Pract. 2012;208(8):489-92. 10.1016/j.prp.2012.05.013 22770943

[16] Dutenhefner SE, Marui S, Santos AB, de Lima EU, Inoue M, Neto JS, et al. BRAF: a tool in the decision to perform elective neck dissection? Thyroid. 2013;23(12):1541-6. 10.1089/thy.2012.0304 23186006

[17] Penna GC, Pestana A, Cameselle JM, Momesso D, de Andrade FA, Vidal APA, et al. TERTp mutation is associated with a shorter progression free survival in patients with aggressive histology subtypes of follicular-cell derived thyroid carcinoma. Endocrine. 2018;61(3):489-98. 10.1007/s12020-018-1642-0 29948935

[18] Pinheiro Dos Santos MJC, Bastos AU, da Costa VR, Delcelo R, Lindsey SC, Colozza-Gama GA, et al. LIMD2 Is Overexpressed in BRAF V600E-Positive Papillary Thyroid Carcinomas and Matched Lymph Node Metastases. Endocr Pathol. 2018;29(3):222-30. 10.1007/s12022-018-9526-7 29560564

[19] da Silva RC, de Paula HS, Leal CB, Cunha BC, de Paula EC, Alencar RC, et al. BRAF overexpression is associated with BRAF V600E mutation in papillary thyroid carcinomas. Genet Mol Res. 2015;14(2):5065-75. 10.4238/2015.May.12.9 26125698

[20] Lutz BS, Leguisamo NM, Cabral NK, Gloria HC, Reiter KC, Agnes G, et al. Imbalance in DNA repair machinery is associated with BRAFV600E mutation and tumour aggressiveness in papillary thyroid carcinoma. Mol Cell Endocrinol. 2018;472:140-8. 10.1016/j.mce.2017.12.004 29229408

[21] Kim HJ, Park HK, Byun DW, Suh K, Yoo MH, Min YK, et al. Iodine intake as a risk factor for BRAF mutations in papillary thyroid cancer patients from an iodine-replete area. Eur J Nutr. 2018;57(2):809-15. 10.1007/s00394-016-1370-2 28258306

[22] Campos Rde O, Reboucas SC, Beck R, de Jesus LR, Ramos YR, Barreto Idos S, et al. Iodine Nutritional Status in Schoolchildren from Public Schools in Brazil: A Cross-Sectional Study Exposes Association with Socioeconomic Factors and Food Insecurity. Thyroid. 2016;26(7):972-9. 10.1089/thy.2015.0448 27184190

[23] Veiga LH, Neta G, Aschebrook-Kilfoy B, Ron E, Devesa SS. Thyroid cancer incidence patterns in São Paulo, Brazil, and the U.S SEER program, 1997-2008. Thyroid. 2013;23(6):748-57. 10.1089/thy.2012.0532 PMC3675840 23410185

[24] Ito Y, Miyauchi A. Prognostic factors and therapeutic strategies for differentiated carcinomas of the thyroid. Endocr J. 2009;56(2):177-92. 10.1507/endocrj.k08e-166 18703852

[25] Shen X, Zhu G, Liu R, Viola D, Elisei R, Puxeddu E, et al. Patient Age-Associated Mortality Risk Is Differentiated by BRAF V600E Status in Papillary Thyroid Cancer. J Clin Oncol. 2018;36(5):438-45. 10.1200/JCO.2017.74.5497 PMC5807010 29240540

[26] Vaisman F, Corbo R, Vaisman M. Thyroid carcinoma in children and adolescents-systematic review of the literature. J Thyroid Res. 2011;2011:845362. 10.4061/2011/845362 PMC3166725 21904689

[27] Nikiforova MN, Ciampi R, Salvatore G, Santoro M, Gandhi M, Knauf JA, et al. Low prevalence of BRAF mutations in radiation-induced thyroid tumours in contrast to sporadic papillary carcinomas. Cancer Lett. 2004;209(1):1-6. 10.1016/j.canlet.2003.12.004 15145515

[28] Liu D, Hu S, Hou P, Jiang D, Condouris S, Xing M. Suppression of BRAF/MEK/MAP kinase pathway restores expression of iodide-metabolizing genes in thyroid cells expressing the V600E BRAF mutant. Clin Cancer Res. 2007;13(4):1341-9. 10.1158/1078-0432.CCR-06-1753 17317846

[29] Hiromatsu Y, Satoh H, Amino N. Hashimoto's thyroiditis: history and future outlook. Hormones (Athens). 2013;12(1):12-8. 10.1007/BF03401282 23624127

[30] Hanahan D, Weinberg RA. Hallmarks of cancer: the next generation. Cell. 2011;144(5):646-74. 10.1016/j.cell.2011.02.013 21376230

[31] Colotta F, Allavena P, Sica A, Garlanda C, Mantovani A. Cancer-related inflammation, the seventh hallmark of cancer: links to genetic instability. Carcinogenesis. 2009;30(7):1073-81. 10.1093/carcin/bgp127 19468060

[32] Russell JP, Shinohara S, Melillo RM, Castellone MD, Santoro M, Rothstein JL. Tyrosine kinase oncoprotein, RET/PTC3, induces the secretion of myeloid growth and chemotactic factors. Oncogene. 2003;22(29):4569-77. 10.1038/sj.onc.1206759 12881713

[33] Borrello MG, Alberti L, Fischer A, Degl'innocenti D, Ferrario C, Gariboldi M, et al. Induction of a proinflammatory program in normal human thyrocytes by the RET/PTC1 oncogene. Proc Natl Acad Sci U S A. 2005;102(41):14825-30. 10.1073/pnas.0503039102 PMC1253545 16203990

[34] Zhou D, Li Z, Bai X. BRAF (V600E) and RET/PTC Promote Proliferation and Migration of Papillary Thyroid Carcinoma Cells In Vitro by Regulating Nuclear Factor-κB. Med Sci Monit. 2017;23:5321-9. 10.12659/MSM.904928 PMC5688787 29117154

[35] Muzza M, Degl'Innocenti D, Colombo C, Perrino M, Ravasi E, Rossi S, et al. The tight relationship between papillary thyroid cancer, autoimmunity and inflammation: clinical and molecular studies. Clin Endocrinol (Oxf). 2010;72(5):702-8. 10.1111/j.1365-2265.2009.03699.x 20447069

[36] Marotta V, Guerra A, Zatelli MC, Uberti ED, Di Stasi V, Faggiano A, et al. BRAF mutation positive papillary thyroid carcinoma is less advanced when Hashimoto's thyroiditis lymphocytic infiltration is present. Clin Endocrinol (Oxf). 2013;79(5):733-8. 10.1111/cen.12194 23469895

[37] Tuttle RM, Haugen B, Perrier ND. Updated American Joint Committee on Cancer/Tumor-Node-Metastasis Staging System for Differentiated and Anaplastic Thyroid Cancer (Eighth Edition): What Changed and Why? Thyroid. 2017;27(6):751-6. 10.1089/thy.2017.0102 PMC5467103 28463585

[38] Quiros RM, Ding HG, Gattuso P, Prinz RA, Xu X. Evidence that one subset of anaplastic thyroid carcinomas are derived from papillary carcinomas due to BRAF and p53 mutations. Cancer. 2005;103(11):2261-8. 10.1002/cncr.21073 15880523

[39] Hou P, Liu D, Xing M. Genome-wide alterations in gene methylation by the BRAF V600E mutation in papillary thyroid cancer cells. Endocr Relat Cancer. 2011;18(6):687-97. 10.1530/ERC-11-0212 PMC3346957 21937738

